# Age-Stratified Long-Term Outcomes of Immune Checkpoint Inhibitors for Stage IV Melanoma and NSCLC in The Netherlands: A Population-Based Study

**DOI:** 10.3390/cancers18122019

**Published:** 2026-06-22

**Authors:** Eline G. M. Steenhuis, Lieke M. van Disseldorp, Femke J. C. Jacobs, Nathalie van Schayk, Karijn Suijkerbuijk, Marieke Louwman, Julia N. S. d’Hooghe, Ronald A. M. Damhuis, Wouter H. van Geffen

**Affiliations:** 1 Department of Pulmonology, Isala, Dokter van Heesweg 2, 8025 AB Zwolle, The Netherlands; 2 Department of Research and Development, Netherlands Comprehensive Cancer Organisation (IKNL), Rijnkade 5, 3511 LC Utrecht, The Netherlands; 3 Department of Information and Advice, Netherlands Comprehensive Cancer Organisation (IKNL), Rijnkade 5, 3511 LC Utrecht, The Netherlands; 4 Department of Oncology, University Medical Centre Utrecht, Utrecht University, Heidelberglaan 100, 3584 CX Utrecht, The Netherlands; 5 Department of Pulmonology, Onze Lieve Vrouwen Gasthuis, Oosterpark 9, 1091 AC Amsterdam, The Netherlands; 6 Department of Pulmonology, Radboud University Medical Centre, Geert Grooteplein Zuid 10, 6525 GA Nijmegen, The Netherlands

**Keywords:** immunotherapy, elderly, NSCLC, melanoma

## Abstract

**Background:** Immune checkpoint inhibitors (ICIs) are standard treatment for melanoma and non-small cell lung cancer (NSCLC), yet evidence on their effectiveness in older adults remains limited due to underrepresentation in clinical trials. This study assessed long-term, age-stratified outcomes of ICI treatment in real-world clinical practice. **Methods:** This nationwide observational study used data from the Netherlands Cancer Registry on patients with synchronous stage IV melanoma or NSCLC who received first-line ICIs between 2018 and 2023. Melanoma treatments included nivolumab plus ipilimumab or anti-PD-1 monotherapy; NSCLC treatments included pembrolizumab with or without chemotherapy. Primary outcomes were five-year overall survival (5-yr OS) and three-year conditional survival (3-yr CS), stratified by age. **Results:** A total of 11,140 patients were included, consisting of 583 patients with melanoma and 10,557 with NSCLC. In the melanoma population, 5-yr OS was 43.8%. Patients aged ≥ 75 years had a 5-yr OS of 30.8% and a 3-yr CS of 58.7%. In NSCLC treated with pembrolizumab monotherapy, 5-yr OS was 23.1%; among patients aged ≥ 75 years, 5-yr OS was 15.6% and 3-yr CS was 46.6%. Pembrolizumab combined with chemotherapy resulted in a 5-yr OS of 14.6%, with corresponding 5-yr OS of 8.4% and 3-yr CS of 35.5% in patients aged ≥ 75 years. **Conclusions:** This registry-based analysis suggests that ICI are associated with durable long-term survival in real-world patients with stage IV melanoma or NSCLC, including selected older adults. These findings are in line with outcomes of clinical trials, but further research is needed on predictors of ICI effectiveness in the older population.

## Simple Summary

Patients with stage IV melanoma or non-small cell lung cancer are regularly treated with immunotherapy, either as monotherapy or combined with other types of treatment. A large proportion of these patients are aged 75 and above. However, it is still unclear how well immunotherapy works in older patients, because they are often not included in large clinical studies. This study describes the survival outcomes of patients treated with immunotherapy in everyday medical practice. The results show that older patients who respond well to immunotherapy can live for a long time after treatment. Future studies should aim to find better ways to predict which older patients will benefit most from immunotherapy. This may include looking at a person’s overall health and physical condition, rather than just their actual age in years.

## 1. Introduction

Immunotherapy has become a cornerstone in the treatment of various solid tumours across different stages of disease progression. Its introduction has transformed the therapeutic landscape for patients with melanoma and non-small cell lung cancer (NSCLC) [1,2,3,4] with markedly improved survival outcomes [3,4].

The efficacy of ICIs in patients with stage IV melanoma and NSCLC has been established through numerous large-scale clinical trials [5,6,7,8,9,10,11,12,13,14]. Reported five-year overall survival (5-yr OS) for patients with stage IV melanoma treated with nivolumab plus ipilimumab, nivolumab monotherapy, or pembrolizumab monotherapy is 52%, 44% and 39%, respectively [10,11]. Five-yr OS for patients with stage IV NSCLC treated with pembrolizumab monotherapy is associated with Programmed Death Ligand-1 (PD-L1) Tumour Proportion Score (TPS) and ranges between 16.6 and 21.9% [14]. Five-yr OS of patients with advanced NSCLC treated with pembrolizumab and chemotherapy are 18.4% for squamous NSCLC [12] and 19.4% for non-squamous NSCLC [13].

These studies, however, have mainly included highly selected populations due to strict inclusion and exclusion criteria. As a result, patients frequently seen in routine clinical practice—such as older patients and individuals with poor World Health Organisation performance status (WHO PS)—are persistently underrepresented [15,16]. Real-world data on long-term survival outcomes in these patient populations are also scarce.

These data gaps are particularly relevant given that the incidence of both melanoma and NSCLC increases with age [15,16,17]. More than 60% of advanced NSCLC diagnoses occur in individuals aged 65 years or older, and approximately one-third is aged ≥ 75 years [15,17,18].

Furthermore, the concept of immunosenescence—the gradual deterioration of the immune system associated with ageing—raises important concerns regarding the efficacy of ICIs in older patients. This age-related decline, partly characterised by reduced T-cell function, could potentially impair the therapeutic effectiveness of ICIs [19].

The growing recognition of this evidence gap has led to increased efforts to collect data on these underrepresented populations. A meta-analysis [20] combined information from 12 randomised studies to evaluate the efficacy of ICIs among patients aged ≥ 75 years. Of the included studies, nine involved patients with NSCLC, while the remaining studies included patients with melanoma, small cell lung cancer (SCLC) or renal cell cancer. In this pooled analysis, the survival benefit of ICIs was only marginally significant (HR 0.84, 95% CI 0.70–1), and no pooled results were available by cancer type. A subgroup analysis of trials evaluating first-line ICI therapy only (NSCLC, *n* = 4; melanoma, *n* = 1; SCLC, *n* = 1) demonstrated a significant overall survival benefit (HR 0.77, 95% CI 0.61–0.96). However, substantial heterogeneity was observed across this small number of studies. More recently, a systematic review and meta-analysis [21] assessed the efficacy and safety of ICIs in patients aged ≥ 65 years with NSCLC, using data from both randomised controlled trials (RCTs) and real-world studies. Pooled RCT data showed a significant improvement in overall survival for patients treated with ICI monotherapy compared with chemotherapy, but this benefit was not observed in patients aged ≥ 75 years. In contrast, real-world studies did not demonstrate age-related differences in outcomes. This discrepancy may be explained by selection bias in RCTs and the underrepresentation of patients aged ≥ 75 years.

In conclusion, existing evidence is limited to small analyses with considerable heterogeneity in included populations. Inconsistencies in age cut-offs across studies further hinder the availability of robust data specifically concerning patients aged 75 and above [15,16,20,21].

Data from population-based registries can provide information on large patient groups and describe outcomes of treatment strategies for elderly patients, particularly in the context of a rapidly ageing population.

This observational study aims to generate nationwide real-world data on age-stratified long-term clinical outcomes for patients with stage IV melanoma and NSCLC, treated with ICIs.

## 2. Patients and Methods

### 2.1. Patients

For this study, we queried the Netherlands Cancer Registry (NCR) for adult patients with organ-metastatic skin melanoma or melanoma of unknown primary (TNM metastasis stages M1b/c/d) or NSCLC (TNM stage IV) who were diagnosed in the period of 1 January 2018 to 31 December 2023 and who received first-line treatment with ICIs. Only patients with synchronously metastatic disease were included.

Eligible patients were aged 18 years or older, with any WHO PS. Patients with specific driver mutations for whom no TKI option was available in first line were included.

Patients with melanoma received either dual immunotherapy with nivolumab and ipilimumab or monotherapy with anti-PD-1 checkpoint inhibitors. Baseline characteristics and survival outcomes were analysed for the melanoma group as a whole. Patients with NSCLC were treated either with pembrolizumab monotherapy or pembrolizumab in combination with chemotherapy (pemetrexed, paclitaxel, cisplatin and/or carboplatin). These patient groups were analysed separately.

We excluded patients who previously or simultaneously received other forms of systemic therapy and patients who did not initiate the agents of the combination therapy on the same day.

### 2.2. Data Sources

Patient and tumour characteristics as well as survival data were obtained from the NCR which is maintained by the Netherlands Comprehensive Cancer Organisation (IKNL).

The NCR collects data on all patients with de novo cancer diagnosed in the Netherlands following notification of newly diagnosed malignancies by hospital diagnoses and the national automated pathological archive (Palga). Data on patient and tumour characteristics as well as diagnostic information and first-line treatment were extracted from the medical records in all Dutch hospitals by trained NCR data managers. Stage information was registered according to the eighth edition of the Union for International Cancer Control (UICC) TNM classification at the moment of first diagnosis. Affected organs with distant metastases were recorded separately, allowing distinction between brain and liver metastases.

Information about vital status was obtained by annual linkage with data from the Dutch Civil Registry, with follow-up data complete up to 31 January 2025. Data on toxicity, treatment response, disease progression, subsequent lines of treatment, and cause of death were not available.

### 2.3. Analyses

Patients were included at the start of first-line ICI treatment and followed until death or until 31 January 2025. The primary endpoints were 5-yr OS, and three-year conditional survival (3-yr CS) among patients who had survived two years from treatment initiation. Conditional survival reflects the probability of surviving an additional period of time, given that a patient has already survived a specified duration since diagnosis or treatment start. In oncological research, this dynamic survival measure is of particular interest because prognosis changes over time and mortality risk decreases over time [22]. Our study shows the probability of surviving another 3 years for those patients who already survived the first two years after the start of treatment.

Secondary outcomes of this study include the relative survival of the study groups, using the Ederer II method [23]. Relative survival represents the survival of patients relative to that expected in the general population with similar demographic characteristics. Accounting for background mortality provides an estimate of disease-associated excess mortality without requiring information on cause of death.

Patient characteristics were tabulated by tumour and treatment type. Associations between WHO PS and age were tabulated and tested using chi-square tests.

Overall survival and conditional survival were calculated from the day of starting ICI till death or the day of censoring (31 January 2025) and reported with 95% confidence intervals (95% CI). To facilitate the interpretation of survival estimates over time, the number of patients at risk is shown. Median follow-up was estimated using the reverse Kaplan–Meier method. Survival analyses were stratified by tumour type and age. For patients with NSCLC, analyses were also stratified by treatment regimen. Due to the descriptive nature of this study, we refrained from multivariable analyses.

## 3. Results

A total of 11,140 patients were included in this study. Amongst those, 583 patients had synchronous stage IV melanoma and 10,557 patients had stage IV NSCLC. Patient characteristics by tumour type and treatment group are shown in Table 1.

### 3.1. Melanoma Cohort

Slightly more than half of the patients with melanoma were treated with nivolumab and ipilimumab (324, 55.6%). About one-quarter of patients were aged 75 and above (Table 1). The majority of patients had WHO PS 0-1, only 8.8% had WHO 2 or higher. Liver metastases were present in 158 (27.1%) patients and brain metastases were present in 198 (34.0%).

### 3.2. NSCLC Cohort

Among the 10,557 patients with stage IV NSCLC included in the study, 3533 received pembrolizumab monotherapy and 7024 were treated with pembrolizumab in combination with chemotherapy. Patients receiving pembrolizumab monotherapy had notably higher PD-L1 TPS compared to those receiving combination therapy. Specifically, 95.5% of patients in the monotherapy group had PD-L1 TPS ≥ 50%, whereas nearly half of the patients in the combination group had PD-L1 TPS < 1% (Table 1).

The proportion of patients aged 75 years or older was lower in the combination therapy group (16.5%) compared to the monotherapy group (24.7%).

In both treatment groups, WHO PS 0 or 1 was most common. However, WHO PS ≥ 2 was more frequently observed in the monotherapy group compared to the combination group (13.8% vs. 8.8%). More than 60% of patients in both groups presented with multiple extrathoracic metastases, with brain and liver metastases being less common compared to melanoma.

### 3.3. Survival Outcomes

**Figure 1:**
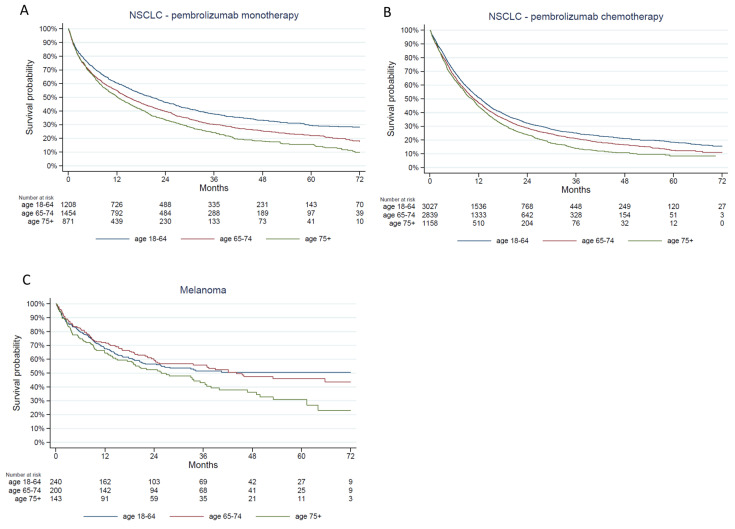
(**A**) Overall survival of patients with NSCLC treated with pembrolizumab monotherapy, stratified by age group. (**B**) Overall survival of patients with NSCLC treated with pembrolizumab plus chemotherapy, stratified by age group. (**C**) Overall survival of patients with melanoma treated with immune checkpoint inhibitors, stratified by age group.

**Figure 2:**
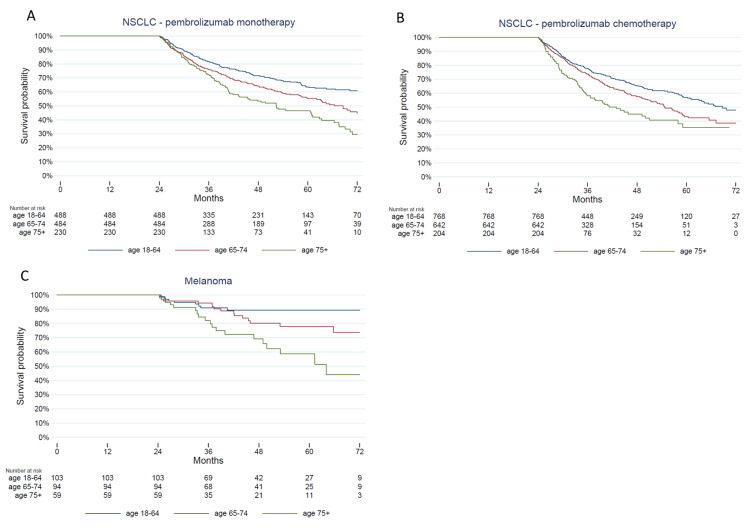
Three-year conditional survival amongst those surviving the first two years after treatment initiation, stratified by age group. (**A**) Patients with NSCLC receiving pembrolizumab monotherapy, (**B**) Patients with NSCLC receiving pembrolizumab plus chemotherapy. (**C**) Patients with melanoma receiving treatment with immune checkpoint inhibitors.

Figure 1 illustrates OS by age group, stratified by disease and, for NSCLC, additionally by treatment group. Figure 2 displays the concomitant 3-yr CS among patients who survived the first two years after treatment. Supplementary Figure S1 depicts the relative survival curves by age group, disease, and for NSCLC by treatment group. Detailed survival outcomes are presented in Table 2.

#### 3.3.1. Melanoma

Median OS was 39.6 months (95% CI 29.0–50.8) and 5-yr OS was 43.8% (95% CI 39.0–48.6). Patients treated with nivolumab and ipilimumab had a 5-yr OS of 45.9% and patients treated with anti-PD1 monotherapy had a 5-yr OS of 41.6%.

Survival outcomes varied by age, with a 5-yr OS of 50.6% in patients under 65 years, 46.0% in patients aged 65–74 and 30.8% in those aged 75 years and above. Three-yr CS amongst those who survived the first two years after initiation of treatment was 89.4%, 77.8% and 58.7%, respectively. Additionally, 5-yr relative survival was 51.7%, 50.9%, and 40.9%, respectively.

During the follow-up period, 49.9% of the patients with melanoma died. The median follow-up of censored observations was 40.6 months (95% CI 38.1–43.3).

#### 3.3.2. NSCLC

In the whole NSCLC cohort, median OS was 12.4 months (95% CI 12.0–12.9) and 5-yr OS was 17.7%. Stratified by age group (from younger to older), 5-yr OS was 21.8%, 16.1% and 11.8%, respectively.

##### NSCLC Cohort Treated with Pembrolizumab Monotherapy

Patients treated with pembrolizumab monotherapy had a median OS of 16.8 months (95% CI 15.6–18.0) and a 5-yr OS of 23.1% (95% CI 21.5–24.8). Five-yr OS stratified by age group was 29.5%, 22.0% and 15.6% from the youngest to the oldest group. Three-yr CS was 63.6%, 55.2% and 46.6%, and 5-yr relative survival was 30.4%, 24.0%, and 20.7% for the respective subgroups.

During the follow-up time, 71.1% of the patients in this cohort died. Median follow-up of censored observations was 50.1 months (95% CI 48.0–51.8).

##### NSCLC Cohort Treated with Pembrolizumab and Chemotherapy

In the combined treatment group, median OS and 5-yr OS were 12.6 months (95% CI 12.2–13.2) and 14.6% (95% CI 13.5–15.8), respectively. Five-yr OS declined with higher age (18.4%, 12.4% and 8.4% for the subgroups). Three-yr CS was 57.0%, 43.2%, and 35.5%, respectively. Corresponding 5-yr relative survival was 19.1%, 14.3%, and 11.5%.

In this cohort, 76.9% of the patients died during the follow-up. The median follow-up of censored observations was 39.1 months (95% CI 37.8–40.3).

#### 3.3.3. Association Between Age and WHO PS

Table 3 shows a clear relationship between age and WHO PS. Older patients, particularly those aged 75 years and above, are more likely to have an impaired performance status (WHO PS ≥ 2). This trend is observed across both the melanoma and NSCLC cohorts, and in patients treated with either monotherapy or combination therapy.

## 4. Discussion

In Dutch real-world clinical practice, a substantial proportion of patients receiving first-line immunotherapy were aged 75 years and older (24.5% in melanoma, 24.7% in NSCLC treated with ICI-monotherapy and 16.5% in NSCLC receiving pembrolizumab-chemotherapy combination). Findings of this study suggest that selected older patients with stage IV melanoma or NSCLC experience durable responses to ICI, resulting in sustained long-term survival in routine clinical practice.

The OS curves demonstrate a high fatality rate during the first two years following treatment initiation, presumably driven by the high proportion of non-responders. The 3-yr CS is notably better, providing insights in prognosis of responders. Among patients aged ≥ 75 years, 3-yr CS was highest for melanoma (58.7%) and remained substantial for NSCLC (46.6% for patients with pembrolizumab monotherapy and 35.5% for patients with pembrolizumab and chemotherapy). These results support the hypothesis that selected older adults can achieve durable survival following ICI-based treatment, but also highlight the importance of individualised treatment selection in older patients.

Across all three treatment groups, outcomes decline with increasing age. Several factors other than immunosenescence may contribute to the observed findings; for instance, older patients more often discontinue treatment early [24], are less likely to receive active treatment when disease progresses, and may be more susceptible to treatment-related toxicity. Furthermore, older adults are more prone to mortality from competing causes.

In addition to OS analyses, relative survival analyses were conducted to compare observed mortality in the study population with the expected mortality in the general population matched on age, sex and calendar year. This method provides an estimate of the excess mortality burden associated with lung cancer. While relative survival declined with age, it should be noted that the expected survival was derived from the matched counterparts of a general population. Because patients with NSCLC often have a higher prevalence of smoking-related comorbidities than the general population, part of the excess mortality captured by the relative survival may be attributable to these comorbid conditions rather than NSCLC alone. All age groups demonstrated markedly impaired survival compared with their age-, sex-, and calendar year matched counterparts in the general population, suggesting that melanoma and particularly NSCLC are associated with substantial excess mortality regardless of age.

Similar survival outcomes have been reported in several clinical trials and other real-world studies [24,25,26,27], indicating that elderly patients can derive substantial benefit from ICI therapy. Two recent phase III studies [28,29] evaluated immunotherapy versus chemotherapy in advanced NSCLC patients who are typically underrepresented in trials. Both trials demonstrated survival benefits of immunotherapy and a more favourable safety profile in older patients with adequate functional status. Although all patients with stage IV melanoma or NSCLC receiving first-line ICI (-based) therapy in Dutch hospitals were included in this study, there is still a potential selection bias. Patients and clinicians can refrain from immunotherapy based on clinical factors such as frailty, comorbidity, or geriatric assessment data, potentially ruling out the frailest patients from the study population.

It is important to consider that the ageing process is accompanied by considerable heterogeneity in physiological reserve, comorbidities and organ dysfunction. Chronological age alone is insufficient to capture these factors. Frailty, although more prevalent among older cancer patients, is not exclusive to this group and may better reflect biological age. It is associated with worse outcomes such as treatment-related toxicity, prolonged hospitalisation and mortality [17,30]. While frailty is challenging to define comprehensively, the WHO PS may function as a marker to reflect overall functional capacity. Data on the safety and efficacy of ICI in individuals with poorer WHO PS are often not reported, and patients with a poor WHO PS remain a high-risk group in whom immunotherapy may be less effective or even harmful [29,31].

A major limitation of this study is the lack of detailed clinical information, such as data on toxicity, hospitalisation, quality of life, objective response, progression-free survival, and subsequent lines of treatment. In particular, the absence of toxicity data precludes assessment of the incidence of immune-related adverse events (irAEs). Although it is often assumed that older patients are more susceptible to irAEs—potentially influencing overall survival—available evidence is conflicting, and most studies do not demonstrate a higher risk of irAEs in elderly populations [24,32]. Without these clinical details, survival outcomes should be interpreted with caution by clinicians, as information on treatment tolerability and net benefit is lacking. However, it should be acknowledged that life expectancy at age 75 in the Netherlands remains substantial—11.8 years for men and 13.4 years for women, suggesting that active treatment may be worthwhile.

Given the descriptive nature of the study, multivariable analyses were not performed. As a result, confounding by known prognostic factors such as PD-L1 expression and metastatic burden could not be addressed. The reported survival differences between age groups should therefore be interpreted as descriptive associations and not as evidence that age is an independent prognostic factor. Age and performance status were closely interrelated, but the limited sample size precluded stratified analyses to evaluate independent effects on survival. In addition, patients with WHO performance status >2 likely represent a heterogeneous group with varying levels of mobility and functional impairment. Unfortunately, no detailed data on specific functional status or mobility were available in the registry.

Furthermore, the study population consisted exclusively of patients with synchronous metastatic disease. As metachronous metastases are more common in melanoma, the melanoma population is relatively small. As a consequence, further stratification by treatment type within this population was not feasible which limited the comparability of our findings to other series. Importantly, synchronous metastatic disease has been associated with poorer outcomes following immune checkpoint inhibitor treatment [31], which may have contributed to inferior results in our cohort.

Finally, the absence of a control group receiving other systemic treatment limits the assessment of the effectiveness of immune checkpoint inhibitor treatment, particularly in elderly patients.

A key strength of this study is the use of nationwide real-world data, which enhances the validity of the findings for a more representative population of patients. In addition, the long follow-up period and the addition of 3-yr CS are important strengths of our study.

Future research should focus on identifying clinical and biological markers that predict immunotherapy outcomes. This includes better characterisation of patients who are at increased risk of toxicity, especially those with advanced age or poor performance status. Although this study did not address cancer nanovaccines, future research should investigate the safety, immunogenicity, and clinical efficacy of such approaches specifically in the older population [33]. Importantly, the expanding group of elderly patients should be given the opportunity to receive immunotherapy after proper counselling about the potential risks and benefits.

## 5. Conclusions

In conclusion, this nationwide real-world study shows that selected older adults with stage IV melanoma and NSCLC treated with ICIs can achieve durable long-term survival in routine practice. Better predictors of benefit, including measures of frailty and functional status, are needed to guide treatment decisions.

## Figures and Tables

**Table 1:** Patient characteristics.

		NSCLC Pembrolizumab Monotherapy (*n* = 3533)		NSCLC Pembrolizumab with Chemotherapy (*n* = 7024)		Melanoma (*n* = 583)	
		*n*	%	*n*	%	*n*	%
**Age**	18–64	1208	34.2%	3027	43.1%	240	41.2%
	65–74	1454	41.2%	2839	40.4%	200	34.3%
	75+	871	24.7%	1158	16.5%	143	24.5%
**Sex**	Men	1845	52.2%	3908	55.6%	406	69.6%
	Women	1688	47.8%	3116	44.4%	177	30.4%
**WHO PS**	0	1078	30.5%	2389	34.0%	226	38.8%
	1	1533	43.4%	3051	43.4%	200	34.3%
	2+	487	13.8%	615	8.8%	51	8.8%
	Unknown	435	12.3%	969	13.8%	106	18.2%
**TNM** **M-stage**	1a	774	21.9%	1572	22.4%	-	-
	1b	635	18.0%	1081	15.4%	92	15.8%
	1c	2124	60.1%	4371	62.2%	306	52.5%
	1d	-	-	-	-	185	31.7%
**PD-L1** **score**	<1	18	0.5%	3189	45.4%	*	
	1–49	52	1.5%	2306	32.8%		
	50+	3375	95.5%	1039	14.8%		
	Unknown	88	2.5%	490	7.0%		
**Brain metastases**	Yes	723	20.5%	1348	19.2%	198	34.0%
	No	2810	79.5%	5676	80.8%	385	66.0%
**Liver metastases**	Yes	507	14.4%	1179	16.8%	158	27.1%
	No	3026	85.7%	5845	83.2%	425	72.9%
**Period**	2018–2020	1811	51.3%	2554	36.4%	251	43.1%
	2021–2023	1722	48.7%	4470	63.6%	332	57.0%

* PD-L1 not registered for melanoma. Abbreviations: NSCLC, non-small cell lung cancer; WHO PS, WHO performance status.

**Table 2:** Five-year overall survival and three-year conditional survival *.

	Age	*n*	5-Yr OS %	95% CI	3-Yr CS %	95% CI
**NSCLC** **Pembrolizumab monotherapy**	18–64	1208	29.5%	26.5–32.4%	63.6%	58.3–68.4%
	65–74	1454	22.0%	19.4–24.6%	55.2%	49.6–60.5%
	75+	871	15.6%	12.7–18.7%	46.6%	38.6–54.1%
**NSCLC** **Pembrolizumab with chemotherapy**	18–64	3027	18.4%	16.7–20.3%	57.0%	52.2–61.5%
	65–74	2839	12.4%	10.5–14.4%	43.2%	36.9–49.3%
	75+	1158	8.4%	6.0–11.4%	35.5%	25.2–45.9%
**Melanoma**	18–64	240	50.6%	43.5–57.2%	89.4%	80.5–94.4%
	65–74	200	46.0%	37.7–54.0%	77.8%	65.3–86.3%
	75+	143	30.8%	21.4–40.7%	58.7%	41.0–72.7%

* Conditional on being alive at two years after treatment initiation. Abbreviations: NSCLC, non-small cell lung cancer; OS, overall survival; CI, confidence interval; CS, conditional survival.

**Table 3:** Association between age and WHO PS.

			18–64	65–74	75+	*p*
**NSCLC** **Pembrolizumab monotherapy**	WHO PS	0	453 (37.5%)	427 (29.4%)	198 (22.7%)	*p* < 0.001
		1	492 (40.7%)	637 (43.8%)	404 (46.4%)	
		2+	134 (11.1%)	199 (13.7%)	154 (17.7%)	
		unknown	129 (10.7%)	191 (13.1%)	115 (13.2%)	
**NSCLC** **Pembrolizumab** **with chemotherapy**	WHO PS	0	1109 (36.6%)	935 (32.9%)	345 (29.8%)	*p* < 0.001
		1	1241 (41.0%)	1250 (44.0%)	560 (48.4%)	
		2+	249 (8.2%)	252 (8.9%)	114 (9.8%)	
		unknown	428 (14.1%)	402 (14.2%)	139 (12.0%)	
**Melanoma**	WHO PS	0	116 (48.3%)	69 (34.5%)	41 (28.7%)	*p* = 0.004
		1	65 (27.1%)	76 (38.0%)	59 (41.3%)	
		2+	18 (7.5%)	16 (8.0%)	17 (11.9%)	
		unknown	41 (17.1%)	39 (19.5%)	26 (18.2%)	

Abbreviations: NSCLC, non-small cell lung cancer; WHO PS, WHO performance status.

## Data Availability

The data that support the findings of this study are available from the NCR, which is maintained by the Netherlands Comprehensive Cancer Organisation (IKNL). Restrictions apply to the availability of these data, which were used under licence for the current study and are therefore not publicly available. Researchers may request access to the data through a formal application to the Netherlands Comprehensive Cancer Organisation.

## Supplementary Materials

The following supporting information can be downloaded at: https://www.mdpi.com/article/10.3390/cancers18122019/s1, Figure S1: Relative survival by age group, stratified by tumour type and, for NSCLC, by treatment group.

## Abbreviations

ICI	Immune checkpoint inhibitors
IKNL	Netherlands Comprehensive Cancer Organisation
irAEs	Immune-related adverse events
NCR	Netherlands Cancer Registry
NSCLC	Non-small cell lung cancer
PD-L1	Programmed Death-Ligand 1
RCT	Randomised controlled trial
SCLC	Small cell lung cancer
TPS	Tumour proportion score
UICC	Union for International Cancer Control
WHO PS	World Health Organisation performance status
5-yr OS	5-year overall survival
3-yr CS	3-year conditional survival
