# Supplementary material for: Age-Stratified Long-Term Outcomes of Immune Checkpoint Inhibitors for Stage IV Melanoma and NSCLC in The Netherlands: A Population-Based Study
Source: Cancers (Basel). 2026 Jun 22;18(12):2019. doi: 10.3390/cancers18122019 (PMC13297090; doi:10.3390/cancers18122019)
Supplement: Supplementary file 1 [file cancers-18-02019-s001.zip › cancers-4336379-supplementary.pdf]

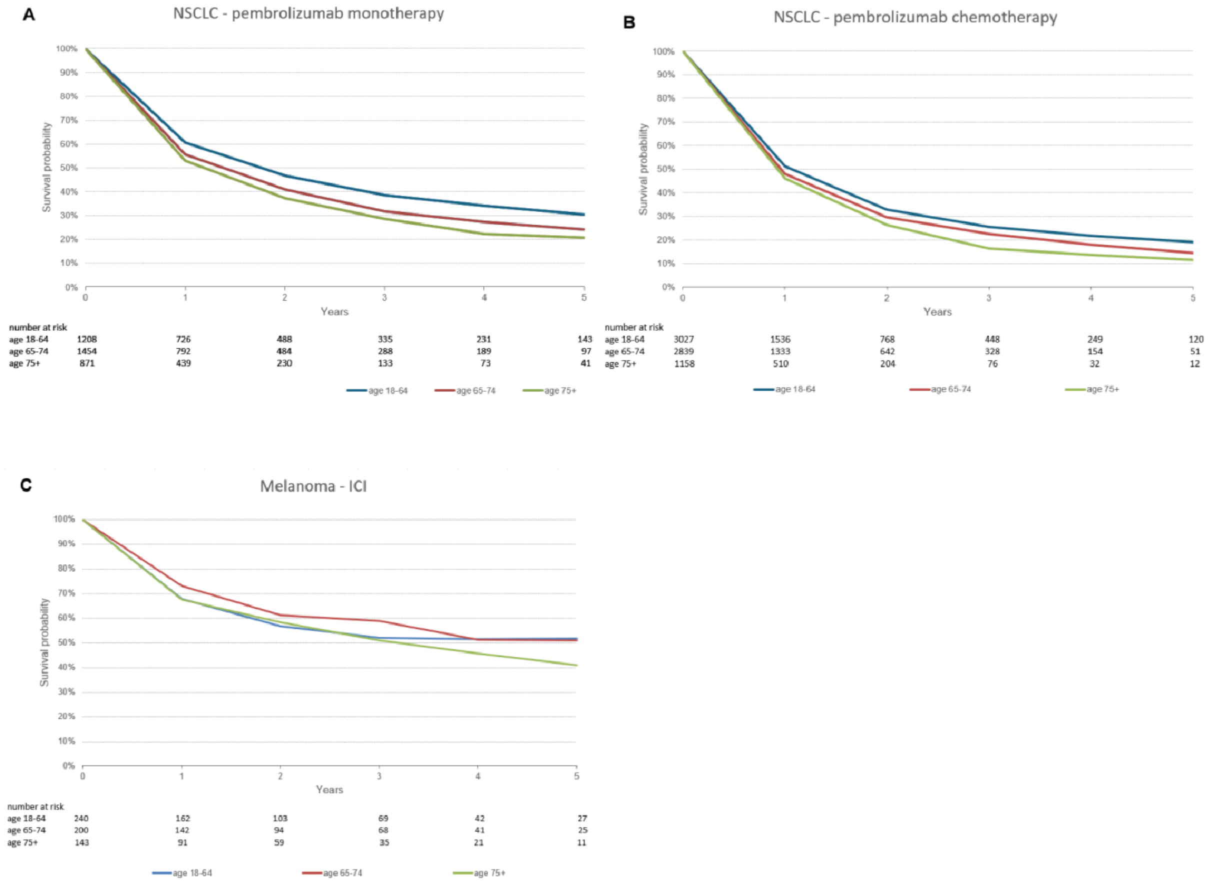

Figure S1. Figure S1. Relative survival by age group, stratified by tumour type and, for NSCLC, by treatment group.
(**A**) Relative survival of patients with NSCLC treated with pembrolizumab monotherapy, stratified by age category, (**B**) Relative survival of patients with NSCLC treated with pembrolizumab and chemotherapy, stratified by age category, (**C**) Relative survival of patients with melanoma, stratified by age category.
